# Integrative analysis of transcriptome and miRNAome reveals molecular mechanisms regulating pericarp thickness in sweet corn during kernel development

**DOI:** 10.3389/fpls.2022.945379

**Published:** 2022-07-25

**Authors:** Caiyun Xiong, Hu Pei, Yahui Zhang, Wenchuang Ren, Ziwei Ma, Yunqi Tang, Jun Huang

**Affiliations:** Guangdong Provincial Key Laboratory of Plant Molecular Breeding, College of Agriculture, South China Agricultural University, Guangzhou, China

**Keywords:** sweet corn, miRNAome, transcriptome, pericarp thickness, kernel development

## Abstract

Pericarp thickness affects the edible quality of sweet corn (*Zea mays* L. *saccharata* Sturt.). Therefore, breeding varieties with a thin pericarp is important for the quality breeding of sweet corn. However, the molecular mechanisms underlying the pericarp development remain largely unclear. We performed an integrative analysis of mRNA and miRNA sequencing to elucidate the genetic mechanism regulating pericarp thickness during kernel development (at 15 days, 19 days, and 23 days after pollination) of two sweet corn inbred lines with different pericarp thicknesses (M03, with a thinner pericarp and M08, with a thicker pericarp). A total of 2,443 and 1,409 differentially expressed genes (DEGs) were identified in M03 and M08, respectively. Our results indicate that phytohormone-mediated programmed cell death (PCD) may play a critical role in determining pericarp thickness in sweet corn. Auxin (AUX), gibberellin (GA), and brassinosteroid (BR) signal transduction may indirectly mediate PCD to regulate pericarp thickness in M03 (the thin pericarp variety). In contrast, abscisic acid (ABA), cytokinin (CK), and ethylene (ETH) signaling may be the key regulators of pericarp PCD in M08 (the thick pericarp variety). Furthermore, 110 differentially expressed microRNAs (DEMIs) and 478 differentially expressed target genes were identified. miRNA164-, miRNA167-, and miRNA156-mediated miRNA–mRNA pairs may participate in regulating pericarp thickness. The expression results of DEGs were validated by quantitative real-time PCR. These findings provide insights into the molecular mechanisms regulating pericarp thickness and propose the objective of breeding sweet corn varieties with a thin pericarp.

## Introduction

Sweet corn (*Zea mays* L. *saccharata* Sturt.) is an endosperm mutant of ordinary corn containing one or more recessive genes (Cox and Dickinson, 1973). It is a vegetable crop cultivated and consumed worldwide because of its unique nutritional value, flavor, and tenderness. The pericarp is a translucent film mainly composed of a cell wall, and variations in its thickness primarily result from programmed cell death (PCD) of pericarp cells during grain development (Li et al., 2021). A thin pericarp has been verified as an important factor in improving the eating quality of sweet corn (Hale et al., 2004). Varieties with a thin pericarp generally exhibit desirable qualities, including improved tenderness, softness, crispness, low residue rate, and good taste (Wu et al., 2020). Sweet corn varieties with high yield and good adaptability have been produced in recent years. However, improvements in edible quality traits, especially through developing varieties with thin pericarps, have not been achieved to date. Therefore, reducing the pericarp thickness has become an important breeding goal to improve the edible quality of sweet corn.

The maize pericarp has also been shown to play an important role in resistance against various pathogens, e.g., *Fusarium moniliforme* and *Fusarium verticillioides*. Previous studies have reported an inverse correlation between pericarp thickness and susceptibility to *Fusarium* attacks. The pericarp of varieties with high or intermediate levels of ear rot resistance was thicker than that of susceptible varieties (Hoenisch and Davis, 1994). Phenylpropanoids and phlobaphenes from the maize pericarp are key resistance factors against *Fusarium* infection, and breeding varieties rich in these two phenolic compounds could be a powerful tool to reduce the accumulation of fumonisin mycotoxin and increase the safety and quality of maize production (Sampietro et al., 2013; Landoni et al., 2020).

Previous studies have shown that maize pericarp thickness has a high narrow-sense heritability involving additive, dominant, and significant epistasis effects (Helm and Zuber, 1972; Wu et al., 2020). A few QTLs regulating pericarp thickness have been identified. Wanlayaporn et al. (2018) investigated the genetic basis underlying pericarp thickness in Thai sweet corn through a recombinant inbred line (RIL) population. They identified a major QTL between bnlg278 and phi128 on chromosome 5, which explained 73.7% of the phenotypic variation in the pericarp thickness of immature sweet corn kernels and 41.6% of the phenotypic variation after maturity. Wu et al. (2020) constructed a BC_4_F_3_ population of 148 families via high-quality genetic linkage mapping containing 3,876 markers and identified 14 QTLs regulating pericarp thickness in sweet corn, and *qPT10*, a stable QTL that can be reproduced in multiple environments, was identified. Park et al. (2013) conducted a QTL analysis for the observed edible quality traits of fresh maize and identified four QTLs regulating pericarp thickness located on chromosomes 4, 5, 8, and 9. Yu et al. (2015b) performed a QTL analysis for pericarp thickness in sweet corn and identified six QTLs affecting pericarp thickness located on chromosomes 2, 3, 5, 6, and 8. Although a few QTLs that regulate pericarp thickness have been reported, no genes have been cloned to date.

With the development of multi-omics (including genomics, epigenomics, and metabolomics), using high-throughput sequencing technology to explore the regulatory network of pericarp formation will comprehensively illuminate the concepts underlying pericarp thickness in sweet corn. Although few genes regulating pericarp thickness in sweet corn have been reported, several genes have also been identified in other crops, particularly tomato. Ma et al. (2014) elucidated the function of *SlNAC*1, which is involved in fruit ripening, and found that overexpression plants reduced ethylene emission by downregulating ethylene biosynthetic genes; however, fruit firmness and pericarp thickness were also reduced in the process. Su et al. (2014) found that silencing the auxin transcriptional repressor *Sl-IAA17* in tomatoes resulted in larger fruit and thicker pericarp by regulating endoreplication-related cell expansion. Liu et al. (2016) revealed that GA- and IAA-mediated miRNAs and their target auxin response factors (ARFs) affect the formation of pericarp cell layers during tomato fruit development, suggesting that phytohormones may regulate pericarp thickness. The loss of function of *SlGPAT6* led to decreased pericarp thickness by altering the expression level of the gene regulating the formation and remodeling of the cuticle and cell wall (Petit et al., 2016), Gupta et al. (2021) revealed that *SlMIR164a* is the major contributor of *sly-miR164* in tomato fruit pericarp and plays a critical role in epicarp expansion. *SlMIR164a* CRISPR/Cas9-derived mutants exhibited thinner fruit pericarp from approximately 10 to 15 DAP than the wild type. Previous studies have collectively revealed that the miRNA-mediated regulation of phytohormone genes may play major role in plant pericarp thickness.

MicroRNAs are approximately 21–23 nucleotide (nt) non-coding RNAs that trigger mRNA cleavage and translational inhibition through homology-mediated pairing with their targets and are involved in plant growth, development, and adaptation to various stresses (Bartel, 2004; Vaucheret, 2006). For example, miR156 targets *SPL10* and *SPL11*, which cause abnormal cell divisions (Nodine and Bartel, 2010). miR159 mediates the cleavage of *MYB33* and *MYB101*, and these two positive regulators of ABA responses were found to play a vital role in *Arabidopsis* seed germination (Reyes and Chua, 2007). miR167 targets *ARF6* and *ARF8*, which are involved in the auxin signal transduction pathway and positively regulate plant development (Yang et al., 2006; Gutierrez et al., 2009). In conclusion, miRNAs can participate in miRNA networks (such as miRNA-GRFs and miRNA-TCPs) to regulate cell proliferation and organ growth. With the ongoing rapid development of next-generation sequencing technology, novel opportunities that employ multi-omics to characterize quantitative traits in maize have been established. For example, a study employing small RNA and transcriptome sequencing showed that six miRNA–mRNA pairs might play essential roles in the maize response to rice black-streaked dwarf virus infection (Li et al., 2018). Furthermore, He et al. (2019) studied the molecular mechanism of heat stress on maize growth and yield and used spatiotemporal miRNA and transcriptome data to investigate the effects of thermotolerance during the vegetative and reproductive development process, identifying key regulators as potential targets for improving thermotolerance. Collectively, these studies have focused on plant development and stress factors. However, the role of miRNAs during pericarp development in sweet corn remains unclear.

In the present study, we performed high-throughput sequencing using two inbred sweet corn lines with different pericarp thicknesses to characterize the miRNAome and transcriptome changes during kernel development (at 15 days, 19 days, and 23 days after pollination). A comprehensive and integrated analysis revealed that miRNA–mRNA interaction pairs were involved in pericarp development and that phytohormone-mediated programmed cell death may determine pericarp thickness. This study elucidates the molecular mechanism regulating pericarp thickness at the transcriptional and posttranscriptional levels in sweet corn and expands our knowledge about the factors affecting sweet corn pericarp thickness.

## Materials and methods

### Plant materials

Two sweet corn inbred lines, M03 (with a thinner pericarp) and M08 (with a thicker pericarp), were used for transcriptome and small RNA sequencing. Both lines were provided by the Laboratory of Sweet Corn Genetic Improvement (College of Agriculture, South China Agricultural University, Guangzhou, China). In the present study, M03 and M08 lines were grown at the Zengcheng Experimental Teaching Base of South China Agricultural University (Guangzhou, Guangdong, China; 113.81°N, 23.13°E) in the autumn of 2017. Three replicates each of M03 and M08 were planted, with each replicate containing 10 plants. The length and the spacing of the rows were 3 m and 70 cm, respectively. Plants were spaced 25 cm apart, and the plot density was 57,000 plants/hm^2^. Crop management, as well as disease and pest control, were carried out according to local recommendations.

### Sample collection for scanning electron microscopy and sequencing

Sample collections were carried out on 15, 19, and 23 days after pollination (DAP). Three self-pollinated ears of each inbred line were sampled and immediately placed on ice at each time point. Ten kernels from the middle of each ear were collected. A blade was used to cut each kernel approximately 3 mm from the top, and tweezers were used to peel off the pericarp, which was transferred into liquid nitrogen for 3 s. Frozen samples were prepared for SEM and sequencing.

A micrometer (HITACHI Regulus 8100, Japan) was used to measure the pericarp thickness of each kernel. The average pericarp thickness of three ears was regarded as the observed value and adopted for subsequent analysis. GraphPad Prism (version 8.0) was used for phenotypic data analysis.

### Library construction

Total RNA was isolated using the SteadyPure Universal RNA Extraction Kit (Vazyme, Nanjing, China) according to the manufacturer’s instructions. The NanoDrop 2000 spectrophotometer (Thermo Fisher Scientific, Waltham, MA, United States), Qubit 2.0 Fluorometer (Life Technologies, Carlsbad, CA, United States), and Agilent 2100 Bioanalyzer System (Agilent Technologies, Santa Clara, CA, United States) were used to evaluate the purity (OD_260/280_ ≥1.8; OD_260/230_ ≥1.0), concentration (total RNA ≥250 ng/μL), and integrity (RIN ≥8.0, 28S/18S ≥1.5) of RNA samples to ensure that they were suitable for transcriptome sequencing.

The mRNA (1.5 μg) from each sample was isolated from the total RNA using oligo(dT) magnetic beads (Invitrogen, Carlsbad, CA, United States), and then fragmented and reverse transcribed into cDNA to construct small RNA sequencing libraries. Adapters with a hairpin loop structure were ligated to cDNA molecules and amplified by PCR. RNA-seq libraries were sequenced using the NEBNext Ultra™ RNA Library Prep Kit for Illumina^®^ (NEB, Ipswich, MA, United States) according to the manufacturer’s instructions.

The RNA bands of approximately 18–30 nt in length were isolated, and then linked to 5′ and 3′ adapters and reverse transcribed to cDNA using the NEBNext^®^ Ultra-small RNA Sample Library Prep Kit (NEB, Ipswich, MA, United States) to construct miRNA-seq libraries. The cDNA fragments were enriched by PCR amplification. Polyacrylamide gel electrophoresis (PAGE) was used to screen the target electrophoresis fragments, with rubber cutting recycling of the pieces to obtain a small RNA library.

### Deep sequencing

High-throughput sequencing of transcriptome and small RNA were performed by Biomarker Technologies (Beijing, China) using the Illumina HiSeq™ 2500 platform (San Diego, CA, United States). Raw reads were quality checked using the FastQC package (Brown et al., 2017)^1^, and adaptor-polluted reads and low-quality reads were removed. The clean reads were mapped to the maize B73 reference genome (version 4.0)^2^ using hisat2 (Kim et al., 2019)^3^ and stringtie (Pertea et al., 2015)^4^.

Bowtie^5^ (Langmead and Salzberg, 2012) was used to obtain unannotated reads containing miRNA. First, all clean reads were aligned with small RNAs in the Silva^6^, GtRNAdb^7^, Rfam^8^, and Repbase^9^ databases to identify and remove rRNA, scRNA, sonRNA, snRNA, and tRNA, as well as repetitive sequences. Next, all unannotated reads were also aligned with the maize B73 reference genome (version 4.0). Finally, the remaining reads were used to identify known and novel miRNAs using the miRDeep2 software (Friedlander et al., 2012).

### Identification of differentially expressed genes and their functional analysis

Gene expression levels were estimated using fragments per kilobase per million reads (FPKM). The abundance of miRNA was quantified using transcripts per million (TPM) algorithm to normalize expressions. Differentially expressed genes (DEGs) and differentially expressed miRNAs (DEMIs) between different samples were identified using the R package DESeq2^10^ (Love et al., 2014). The gene with a false discovery rate (FDR) ≤0.01 and fold change ≥2 was considered a DEG based on three biological replicates. Similarly, an FDR ≤0.05 and a fold change ≥1.5 was considered DEMI (Cao et al., 2019). Target genes of DEMIs were predicted using TargetFinder^11^ (Bo and Wang, 2005).

Gene ontology (GO) annotation^12^ (Ashburner et al., 2000) and Kyoto Encyclopedia of Genes and Genomes (KEGG) pathway analysis^13^ (Kanehisa and Goto, 2000) were conducted to identify the putative biological functions and pathways of DEGs and the target genes.

### Validation of mRNA sequencing

The SteadyPure Universal RNA Extraction Kit and PrimeScript cDNA Synthesis Kit (both from Vazyme, Nanjing, China) were used for total RNA extraction and cDNA synthesis according to the manufacturer’s instructions. cDNA samples were then diluted 10 times and used as qPCR templates. RT-qPCR was performed using SYBR Green Supermix (Tsingke, Beijing, China) on the StepOnePlus PCR platform (Applied Biosystems 7500). The *ZmCYP* gene was adopted as an internal control for mRNA relative expression analysis. Three independent biological replicates were included in the RT-qPCR analysis. A melting curve analysis was performed to evaluate the specificity of the products. The 2^–ΔΔCT^ method was used to calculate the relative expression level of each sample (Livak and Schmittgen, 2001).

## Results

### Scanning electron microscopy analysis of pericarp

**FIGURE 1:**
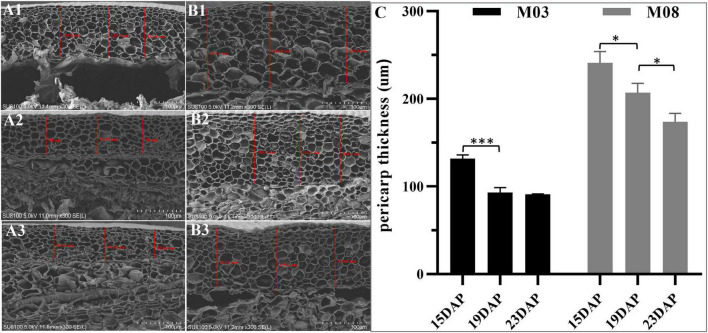
SEM analyses of the changes in pericarp thickness between M03 and M08. **(A)** The pericarp thickness of M03 at 15 **(A1)**, 19 **(A2)**, and 23 **(A3)** DAP. **(B)** The pericarp thickness of M08 at 15 **(B1)**, 19 **(B2)**, and 23 **(B3)** DAP. **(C)** The changes in pericarp thickness in sweet corn lines M03 and M08. Asterisks indicate significant differences between M03 and M08 lines using Student’s *t*-test; *P < 0.05; ***P < 0.001.

The SEM analysis showed that the pericarp in the M03 inbred line was significantly thinner than in the M08 line during kennel development (Figures 1A,B). At 15 DAP, we observed that the average thickness of the pericarp in M03 and M08 samples were 131.79 ± 3.51 μm and 241.02 ± 10.58 μm, respectively. From 15 to 19 DAP, both lines showed a decreasing trend in pericarp thickness; however, M03 displayed a greater proportion of decline than M08 (28.8% in M03 vs. 13.32% in M08). The pericarp thickness in M03 tended to stabilize from 19 to 23 DAP, whereas that in M08 decreased significantly from 207.01 ± 8.67 μm to 173.86 ± 7.72 μm (Figure 1C). SEM analysis also indicated that following the development of the kernel, the cell wall of the pericarp gradually thickened, and the pericarp cells became smaller. Simultaneously, the starch granules in the pericarp cell gradually degraded from 15 to 23 DAP.

### Evaluation of RNA sequencing data

To investigate the molecular mechanisms underlying the regulation of pericarp thickness in sweet corn, we generated a total of 18 mRNA libraries and 18 small RNA sequencing libraries using three biological replicates of sweet corn pericarp. These libraries were sequenced on an Illumina HiSeq™ 2500 platform. Approximately 2.47–2.85 million clean reads corresponding to the six samples were generated, and the Q30 ratio in all samples was greater than 93.38% (Table 1). Hisat2 was used to map these clean reads to the B73 reference genome (B73_RefGen_v4). The results showed that the percentage of clean reads mapped to the reference genome was from 80.65 to 83.74%. After discarding reads that mapped more than twice, approximately 78.32–81.32% of unique mapped reads were used for subsequent analysis. Table 2 shows that 1.14∼1.37 million clean reads were generated from the six samples and that the Q30 ratio in all samples was greater than 98.48%. Bowtie (version 1.1.2) was used to map reads containing miRNA sequencing data to the reference genome, and the match ratio of all samples was over 50.41%. The known miRNAs and novel miRNAs from each sample are summarized in Table 2.

**TABLE 1:** Summary of mRNA sequencing datasets.

Samples	M03-15DAP	M03-19DAP	M03-23DAP	M08-15DAP	M08-19DAP	M08-23DAP
Clean reads	27,388,559	24,866,673	26,557,515	26,558,540	26,303,372	28,534,701
GC content	54.79%	54.96%	55.45%	53.96%	55.39%	54.90%
% ≥Q30	93.43%	93.65%	93.51%	93.38%	94.17%	94.05%
Mapped reads	45,702,163 (83.24%)	40,943,914 (82.33%)	44,209,451 (83.24%)	42,832,464 (80.65%)	44,051,386 (83.74%)	47,485,183 (83.21%)
Unique mapped reads	44,466,465 (81.25%)	39,867,728 (80.16%)	42,945,336 (80.86%)	41,595,125 (78.32%)	42,777,725 (81.32%)	46,041,814 (80.68%)
Multiple mapped reads	1,235,698 (2.25%)	1,076,185 (2.16%)	1,264,116 (2.38%)	1,237,339 (2.33%)	1,273,661 (2.42%)	1,443,369 (2.53%)

**TABLE 2:** Summary of miRNA sequencing datasets.

Samples	M03-15DAP	M03-19DAP	M03-23DAP	M08-15DAP	M08-19DAP	M08-23DAP
Clean-reads	11,815,786	13,041,431	12,789,914	14,134,896	11,362,139	13,724,938
Q30 (%)	99.46	99.01	99.46	99.35	98.79	98.48
Total-reads	7,834,125	8,843,777	7,843,239	10,067,831	8,522,669	10,181,133
Mapped-reads	4,079,058 (52.08%)	4,619,209 (52.20%)	3,981,006 (50.41%)	5,197,523 (51.57%)	4,545,156 (53.35%)	5,347,932 (52.63%)
Known-miRNAs	106	102	106	108	104	110
Novel-miRNAs	120	120	120	121	120	120
Total	226	222	227	229	224	230

### Identification of differentially expressed genes and MiRNAs

**FIGURE 2:**
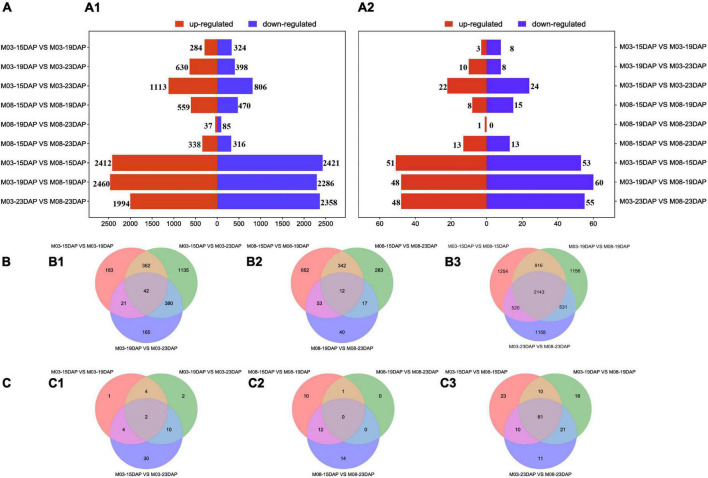
Differentially expressed genes and miRNAs at different stages during kernel development. **(A)** The numbers of upregulated and downregulated DEGs **(A1)** and DEMIs **(A2)** in the different comparison groups. **(B)** Venn diagrams of DEGs in the M03 **(B1)**, M08 **(B2)**, and M03M08 **(B3)** groups. **(C)** Venn diagrams of DEMIs in the M03 **(C1)**, M08 **(C2)**, and M03M08 **(C3)** groups.

The DEGs and DEMIs were identified from nine pairwise combinations of M03, M08, and M03M08 groups using DESeq2 (version 3.14, see text footnote 10) (Figure 2A). The first two groups were composed of three pairwise combinations (M03/M08-15DAP vs. M03/M08-19DAP, M03/M08-15DAP vs. M03/M08-23DAP, and M03/M08-19DAP vs. M03/M08-23DAP), and the M03M08 group was composed of M03-15/19/23 DAP vs. M08-15/19/23 DAP.“M03-15DAP vs. M03-23DAP” had the maximum DEGs (1,919) in the M03 group. “M08-15DAP vs. M08-19DAP” had more DEGs (1,029) than other pairwise comparisons in the M08 group. Moreover, 51, 48, and 48 upregulated and 53, 60, and 55 downregulated DEMIs were identified at 15 DAP, 19 DAP, and 23 DAP, respectively (Figure 2A2). These findings indicate that the two lines may have different regulatory mechanisms that lead to different pericarp thicknesses.

### Construction of the co-expression network between differentially expressed microRNAs and differentially expressed genes

**FIGURE 3:**
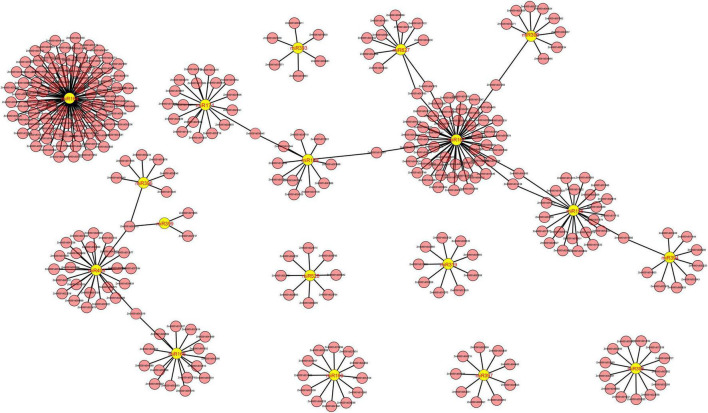
Cystoscope regulatory networks of DEMIs and DEGs. Yellow graphics represent miRNAs, and pink graphics represent genes.

We identified a total of 53, 37, and 152 DEMIs in the M03, M08, and M03M08 groups, respectively (Figure 2C). After removing the duplicated DEMIs, a total of 110 DEMIs were identified, and the corresponding 478 differentially expressed target genes were predicted (Supplementary Table 1). To elucidate the roles of miRNA–mRNA interactions in regulating pericarp thickness during sweet corn pericarp development, regulatory networks of DEMIs and DEGs were constructed (Figure 3). Most of the DEMIs were predicted to target multiple DEGs. For example, zma-miR167 was predicted to target 53 DEGs, *Zm00001d047593* and *Zm00001d047975* were predicted to be the target genes of zma-miR827, and *Zm00001d014138* and *Zm00001d04911* were predicted to be the target genes of miR156. On the other hand, one DEMI may only target single DEGs; for example, *Zm00001d019061* was predicted to be the target gene of zma-miR159, and *Zm00001d031064* was predicted to be the target gene of zma-miR390. Additionally, very few DEGs were targeted by two or more DEMIs; for example, *Zm00001d052112* was predicted to be the target gene of three miRNAs (zma-miR396, zma-miR399, and zma-miR408).

**FIGURE 4:**
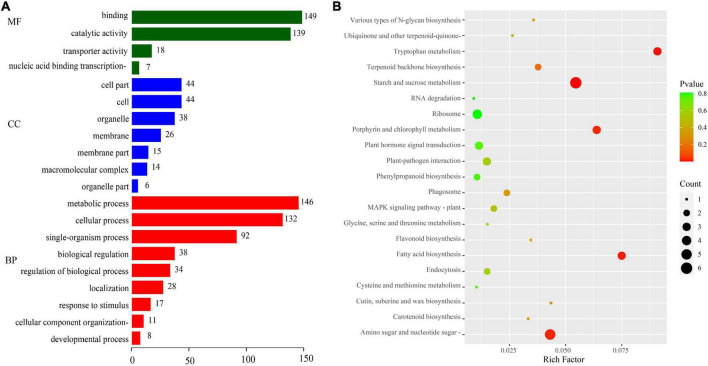
GO annotation and KEGG pathway enrichment analyses of target genes. **(A)** GO annotation. GO terms were classified into three categories: biological process (BP), cellular component (CC), and molecular function (MF). **(B)** KEGG pathway analysis.

We evaluated the potential functions of these target genes by performing GO and KEGG pathway analyses to investigate the regulatory mechanism of DEMIs in sweet corn pericarp thickness during kernel development. The GO enrichment results (Figure 4A) indicated that the most abundant terms for the biological processes (BP) category were “metabolic process” and “cellular process.” The GO terms “catalytic activity” and “binding” had the maximum DEGs for the molecular function (MF) category. In the cellular component (CC) category, “cell,” “cell part,” and “membrane” represented the top terms. The KEGG results showed that “starch and sucrose metabolism” was most significantly enriched (*P* < 0.01), with the maximum number of genes (six DEGs). Next, “tryptophan metabolism,” “fatty acid biosynthesis,” “amino sugar and nucleotide sugar metabolism,” and “porphyrin and chlorophyll metabolism” were significantly enriched (*P* < 0.05). Notably, KEGG results also enriched some pathways, such as “plant hormone signal transduction,” “terpenoid backbone biosynthesis,” “MAPK signaling pathway,” and “glycine, serine, and threonine metabolism” (Figure 4B).

### Functional classification of differentially expressed genes

**FIGURE 5:**
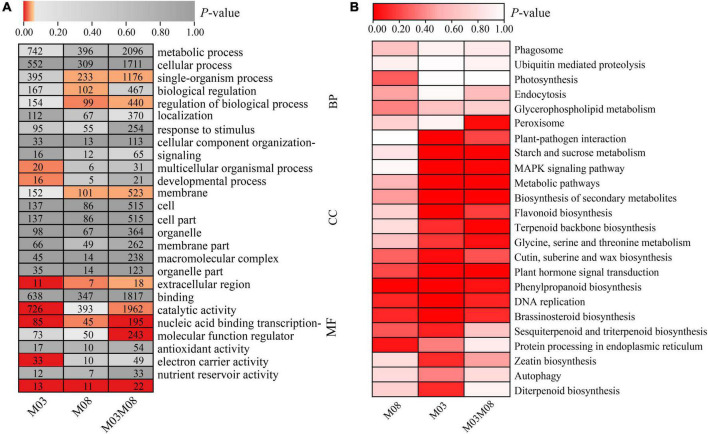
GO and KEGG pathway analysis of DEGs. **(A)** GO annotation of DEGs. In each block of the heatmap, the number represents the gene numbers, and the red coloring represents a *P*-value of ≤0.05. **(B)** KEGG pathway analysis of DEGs. GO terms and KEGG pathway enrichment with *P*-value ≤ 0.05 were identified as significantly enriched.

The GO and KEGG pathway enrichment analyses of DEGs were performed to investigate the biological processes of these DEGs in regulating pericarp thickness during kernel development. A total of 2,443 DEGs from the M03 group, 1,409 DEGs from the M08 group, and 7,678 DEGs from the M03M08 group were obtained (Figure 2B). GO analysis of the DEGs was performed, and GO terms could be classified into three categories: biological process (BP), cellular component (CC), and molecular function (MF; Figure 5A). For the biological processes category, “single-organism process” and “regulation of biological process” were significantly enriched (*P* < 0.05) in the M08 and M03M08 groups, while “multicellular organismal process” and “developmental process” in the M03 group and “biological regulation” in the M08 group were significantly enriched. For the cellular component category, “membrane” was significantly enriched in both the M08 and M03M08 groups, and “extracellular region” was significantly enriched in all three groups, particularly M03. For the molecular function category, “nucleic acid binding transcription factor activity” and “nutrient reservoir activity” were significantly enriched in all three groups. The GO results also showed that the terms of the M03 group were consistently enriched with more genes than those of the M08 group. For example, the number of genes contained in “metabolic processes” was 742 in the M03 group but 396 in the M08 group, suggesting that there may be more genes involved in pericarp thickness regulation in the M03 inbred line.

The KEGG analysis identified the pathways with which DEGs were associated (Figure 5B). Five highly significant pathways, namely, “plant hormone signal transduction,” “phenylpropanoid biosynthesis,” “DNA replication,” “brassinosteroid biosynthesis,” and “cutin, suberin, and wax biosynthesis,” were co-accumulated by all three groups. Additionally, eight pathways were highly significant in the M03 and M03M08 groups, including “starch and sucrose metabolism,” “MAPK signaling pathway,” “terpenoid backbone biosynthesis,” and “glycine, serine, and threonine metabolism,” whereas “sesquiterpenoid and triterpenoid biosynthesis” and “protein processing in endoplasmic reticulum” were highly significant in the M03 and M08 groups.

### Analysis of important pathways regulating the pericarp thickness of sweet corn during kernel development

**FIGURE 6:**
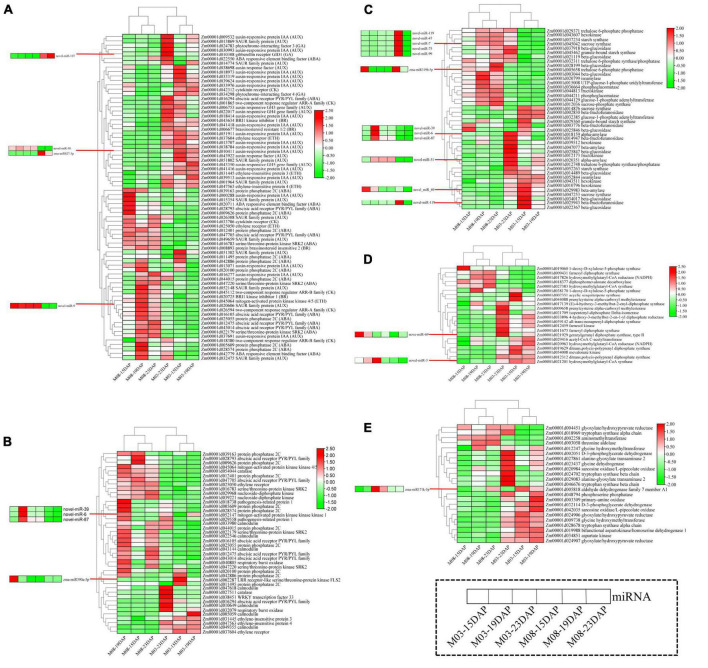
Heatmaps of DEGs and corresponding DEMIs in important pathways. **(A)** Plant hormone signal transduction. **(B)** MAPK signaling pathway. **(C)** Starch and sucrose metabolism. **(D)** Terpenoid backbone biosynthesis. **(E)** Glycine, serine, and threonine metabolism.

Functional analysis of the target genes of DEMIs (Figure 4) and DEGs (Figure 5) during pericarp development identified five important pathways regulating sweet corn pericarp thickness, namely, “plant hormone signal transduction,” “MAPK signaling pathway,” “starch and sucrose metabolism,” “terpenoid backbone biosynthesis,” and “glycine, serine, and threonine metabolism.” The expression patterns of the DEGs involved in these pathways are shown in Figure 6. In the M03 inbred line, DEGs involved in the five pathways showed the same expression patterns at 15 DAP and 19 DAP. In the M08 inbred line, the expression patterns of DEGs enriched in the “MAPK signaling pathway” were similar at 15 DAP and 23 DAP, whereas DEGs in the other four pathways were expressed similarly at 15 DAP and 19 DAP.

The heatmap for the plant hormone signal transduction pathway (Figure 6A) showed that most DEGs related to the abscisic acid (ABA), cytokinin (CK), and ethylene (ETH) signal transduction pathways were downregulated and that DEGs participating in auxin (AUX), gibberellin (GA), and brassinosteroid (BR) signaling pathways were highly expressed in the M03 inbred line. miRNA-mRNA pairs involved in the hormone signal transduction pathway were also identified; *Zm00001d022550* (ABA-responsive element binding factor) was regulated by novel-miR-107 in ABA signal transduction, *Zm00001d043922* (auxin response factor) was regulated by zma-miR827-3p and novel-miR-58 in auxin signal transduction, and *Zm00001d026594* (two-component response regulator ARR-A family) was regulated by novel-miR-9 in cytokinin signal transduction.

The heatmap for the MAPK signaling pathway showed that DEGs exhibited opposite expression patterns in the M03 and M08 lines, i.e., genes that were highly expressed in the M03 inbred line were almost all downregulated in the M08 line (Figure 6B). Surprisingly, 27 genes (64.29%) were highly expressed at 19 DAP in the M08 inbred line, whereas only 7 genes (16.67%) were expressed in the M03 line. The results suggest that genes participating in the MAPK signaling pathway had distinct expression patterns in sweet corn varieties with different pericarp thicknesses, especially at 19 DAP. The target gene of zma-miR390a-5p encodes LRR receptor-like serine/threonine-protein kinase FLS2 (*Zm00001d002287*). zma-miR390a-5p, novel-miR-39, novel-miR-6, and novel-miR-87, which target *Zm00001d052147* (mitogen-activated protein kinase kinase kinase 1), may play central roles in regulating pericarp thickness.

Most genes related to the starch and sucrose metabolism pathways were highly expressed at 23 DAP in the M03 inbred line (23 genes) and M08 inbred line (19 genes) (Figure 6C). Eighteen genes maintained high expression levels at the three stages in M08, of which 10 were only highly expressed at 23 DAP in M03. zma-miR319b-5p targeted *Zm00001d005658* (trehalose 6-phosphate phosphatase), and novel-miR-119 targeted *Zm00001d025943* (beta-fructofuranosidase) and *Zm00001d045042* (sucrose synthase), which is an important regulator in starch and sucrose metabolism.

The heatmap for the terpenoid backbone biosynthesis pathway showed that most genes were highly expressed in the M03 inbred line (Figure 6D). However, five genes, namely, *Zm00001d018377, Zm00001d009431, Zm00001d017826, Zm00001d027383*, and *Zm00001d019060*, were consistently expressed at low levels at the three stages in M03. The target gene of novel-miR-69, which encodes type II geranylgeranyl diphosphate synthase (*Zm00001d021929*), and novel-miR-3, which targets hydroxymethylglutaryl-CoA synthase (*Zm00001d021201*), may be essential in regulating pericarp thickness.

The heatmap for the “glycine, serine, and threonine metabolism” pathway showed that genes were mainly highly expressed in the M03 inbred line and that 23 genes and 20 genes showed high expression levels at 23 DAP and 19 DAP, respectively (Figure 6E). In addition, a gene encoding aminomethyltransferase, *Zm00001d002258*, was highly expressed at all three stages in M03 but always poorly expressed in M08. Here, we identified zma-miR171k-5p, which targeted the aldehyde dehydrogenase family 7 member A1 (*ALDH7A1*) and may be an important regulator in determining pericarp thickness.

### Identification of differentially expressed transcription factors involved in regulating the pericarp thickness of sweet corn

**FIGURE 7:**
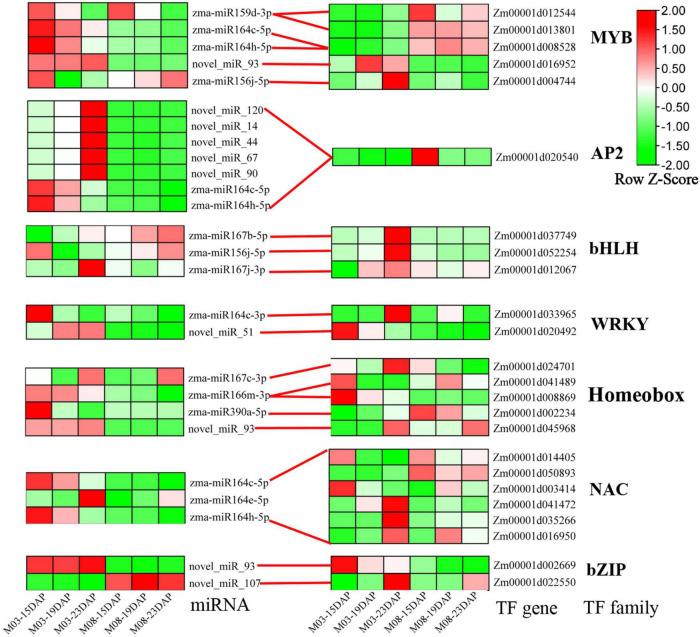
Heatmap of differentially expressed TFs and their corresponding DEMIs.

Transcription factors (TFs) are key components of transcriptional regulatory mechanisms. They are involved in the initiation, regulation, and transcription of genes and may play important roles in plant growth and development. To better understand the role of TFs in regulating the pericarp thickness of sweet corn, we identified differentially expressed TFs (Supplementary Table 2). Here, a total of 626 TFs were discovered from all DEGs. The most abundant TFs belonged to the MYB (64 genes), AP2-EREBP (52 genes), WRKY (47 genes), bHLH (44 genes), Homeobox (42 genes), NAC (36 genes), and bZIP (35 genes) families. The expression patterns of miRNA-TFs modules were also investigated (Figure 7), and most DEMIs were found to target a single differentially expressed gene. Interestingly, certain DEMIs may target one DEG, such as *Zm00001d020540*, an AP2/EREBP transcription factor gene regulated by seven miRNAs. Additionally, some DEMIs targeting many DEGs were identified. For example, six NAC genes (*Zm00001d041472, Zm00001d0144015, Zm00031d035266, Zm00001d016950, Zm00001d003414*, and *Zm00001d050893*) were regulated by zma-miR164c-5p, zma-miR164e-5p, and zma-miR164h-5p.

### Verification of differentially expressed genes via real-time-quantitative PCR

**FIGURE 8:**
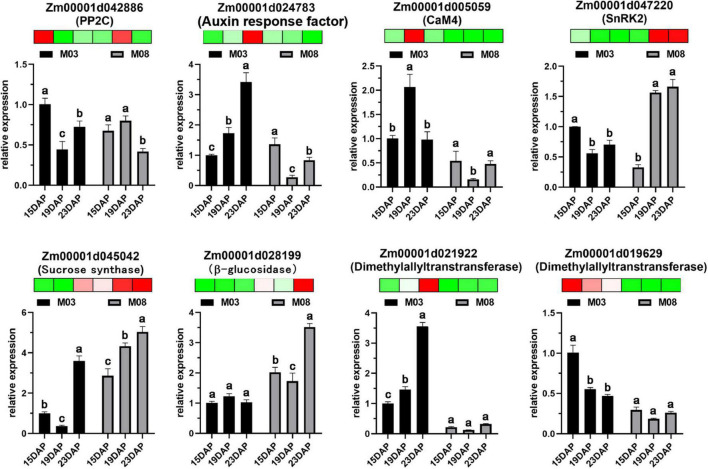
Validation of eight DEGs using RT-qPCR. Error bars represent the standard deviation of three replicates. Heatmaps were generated using transcriptome data; red indicates high expression levels, and green indicates low expression levels. Lowercase letters above the columns represent statistical differences with the same inbred line (*P* < 0.05).

The expression levels of DEGs from the transcriptomic sequencing data were verified by RT-qPCR (Figure 8). Eight DEGs were selected to validate the RNA-seq expression profiles from different pericarp development processes. Primers used for RT-qPCR analysis are listed in Supplementary Table 3. The genes included *Zm00001d042886* (PP2C) and *Zm00001d038698* (auxin response factor), which were involved in ABA and AUX signal transduction in plant hormone signal transduction. *Zm00001d005059* (CaM4) and *Zm00001d047220* (SnRK2) participated in the MAPK signaling pathway. *Zm00001d045042* (sucrose synthase) and *Zm00001d028199* (β-glucosidase) were essential enzymes for starch and sucrose metabolism. *Zm00001d021922* (dimethylallyltranstransferase) and *Zm00001d019629* (dimethylallyltranstransferase) played important roles in terpenoid backbone biosynthesis. The expression levels of the eight selected DEGs were consistent with the RNA-seq data, confirming the reliability of the data.

## Discussion

### Changes in sweet corn pericarp thickness during kernel development

The pericarp of the sweet corn kernel is formed via the development of the ovary wall, which wraps the outer layer of the caryopsis and protects the endosperm and embryo. Our results showed that pericarp thickness decreased from 15 to 23 days after pollination. It was previously suggested that pericarp development is a typical process involving programmed cell death (PCD; Zhou et al., 2009). PCD in plants occurs via autophagy, a ubiquitous and evolutionarily conserved degradation pathway found in all eukaryotes, by which cells degrade and recycle cytoplasmic material to maintain normal cell differentiation and development (Yoshimoto, 2012). Autophagy is triggered by plant hormones (such as ETH, BR, ABA, and CK), and the “death signal” may be transduced by hormonal signaling pathways (Kuriyama and Fukuda, 2002; Gadjev et al., 2008). Pericarp cells produce large amounts of α-amylase, which transforms into sugar and is transported to the endosperm as the substrate for starch synthesis (Radchuk et al., 2018) before undergoing autophagy. Consistently, we found that “autophagy” was identified during KEGG analysis (Figure 5B).

Additionally, we observed that the cell wall gradually thickened, the cells became smaller and more compact, and starch granules in the pericarp cells gradually degraded from 15 to 23 days after pollination. The pericarp of sweet corn is mainly composed of cellulose, which surrounds the entire seed, protects the seed from external mechanical damage, and prevents the invasion of pests and diseases. During the later stage of kernel development, the pericarp cells stop growing, the volume remains unchanged, and the cells squeeze against each other and are arranged more densely (Yue et al., 2012). At this time, the active products of the protoplast accumulate and thicken the primary cell wall, reducing the cell cavity and cell wall ductility, thereby enhancing the mechanical support of the cell (Bourquin et al., 2002).

### Integration analysis of differentially expressed microRNAs and differentially expressed genes

The development of sequencing technology has facilitated a clearer understanding of the complex mechanisms regulating sweet corn pericarp thickness at the whole genome level. In the present study, a total of 110 DEMIs and 478 differentially expressed target genes were identified. zma-miR164 (90 genes), zma-miR167 (53 genes), zma-miR156 (25 genes), zma-miR408 (25 genes), and zma-miR171 (18 genes) belong to miRNA families with the largest number of predicted target genes, many of which are transcription factors. For example, an AP2-like ethylene transcription factor (*Zm00001d020540*) and seven NAC-transcription factors were regulated by zma-miR164. In addition, six ARF-transcription factors were regulated by zma-miR167. zma-miR156 regulates seven SPL-transcription factors, zma-miR408 regulates *Zm00001d052112* (GRF-transcription factor), and zma-miR171 regulates four GRAS-transcription factors. Recently, studies have shown that miRNAs regulate or depend on genes related to plant hormone signal transduction to participate in plant growth and development. zma-miR164 bound to MYB and the NAC-transcription factor to regulate the growth of maize plants under drought conditions in an ABA-dependent manner (Liu et al., 2019). zma-miR164 may also regulate its targets in response to auxin signaling, thereby regulating internode elongation and development under the maize ear (Zhao et al., 2016). The auxin-responsive factor ARF8, which is regulated by miR167, participated in the development of young tissues, especially shoot apices and flower elements (Glazinska et al., 2014). The increased levels of three closely related miR156-targeted Arabidopsis SPL-transcription factors (SPL2, SPL10, and SPL11) suppressed root regeneration with age by inhibiting wound-induced auxin biosynthesis (Ye et al., 2020). In addition, both miR156 and SPLs (SPL3, SPL9, and SPL10) were responsive to auxin signaling, suggesting that the miR156/SPL modules might be involved in determining the proper timing of lateral root developmental progression (Yu et al., 2015a). Additionally, miR408 bound to PIF1 (PHYTOCHROME INTERACTING FACTOR 1) to regulate the balance between GA and ABA during Arabidopsis seed germination (Jiang et al., 2021), and the miR171-SlGRAS24 module participated in a series of developmental processes by modulating gibberellin and auxin signaling (Huang et al., 2017). Thus, these miRNA families may regulate pericarp thickness by participating in phytohormone signaling pathways.

### The important pathways regulating the pericarp thickness of sweet corn during kernel development

During kernel development in sweet corn, cell proliferation and rapid cell expansion are regulated by endogenous plant hormones (Ivakov and Persson, 2013) and gene expression involved in apoplast acidification, cell wall relaxation, sugar lysis, water transport, and cell wall biosynthesis (Pielot et al., 2015). Our study analyzed the regulation mechanism between different lines during the kernel development process and found that “plant hormone signal transduction,” “MAPK signaling pathway,” “starch and sucrose metabolism,” “terpenoid backbone biosynthesis,” and “glycine, serine, and threonine metabolism” may play important roles in regulating changes in the pericarp thickness.

Kernel development is dependent on a robust but highly controlled cell death activation, and most plant “death signal” is triggered by plant hormones. It has been demonstrated that high CK levels induce PCD in plant cells (Carimi et al., 2003). The phytohormone GA triggers a series of events in cereal aleurone cells that lead to PCD (Xie et al., 2014; Hou et al., 2017). Autophagy-related gene transcripts and autophagosome formation in tomatoes are triggered by enhanced levels of BZR1-dependent BR (Wang et al., 2019; Chi et al., 2020). Although auxin appears to suppress cell death, there is growing evidence that it can promote PCD events, most likely by stimulating ETH biosynthesis (Kacprzyk et al., 2021). Cereal endosperm undergoes PCD during its development, a process controlled in part by ETH. At the same time, a balance between ABA and ETH establishes the appropriate occurrence and progression of programmed cell death during maize endosperm development (Young and Gallie, 2000). Furthermore, accumulation of ETH and GA and reduction of ABA levels in rice internodes favored the induction of early PCD in epidermal cells covering adventitious root primordia, which could prevent injury to the growing roots under flooding conditions (Steffens and Sauter, 2005). In our study, most of the DEGs related to ABA, CK, and ETH signal transduction were highly expressed in the M08 inbred line (thicker pericarp), especially at 19 DAP and 23 DAP. This finding suggests that these three hormones may be actively involved in PCD in the thick pericarp variety to degrade pericarp cells. Most of the DEGs related to signal transduction of AUX, GA, and BR were highly expressed in the M03 line. GA and AUX might induce ABA- or ETH-mediated PCD in pericarp cells, and BR might be involved in regulating pericarp cell thickness in thin-peel varieties in a BZR1-dependent manner.

Mitogen-activated protein kinase (MAPK) modules play critical roles in the transduction of environmental and developmental signals through the phosphorylation of downstream signaling targets in all eukaryotic cells (Xu and Zhang, 2015). The MAPK cascade pathway regulates phragmoplast dynamics during cytokinesis in plants (Nishihama et al., 2001; Soyano et al., 2003). The timely activation of multiple regulatory pathways, including the AtNACK1/HINKEL kinesin-induced MAPK cascade, was essential to cytokinesis (Takahashi et al., 2010; Saito et al., 2015). Crosstalk has been found between the MAPK cascade and the phytohormone signaling pathway. The available data suggest that MAPK cascades are involved in some ABA responses, and several MAPK phosphatases (PP2Cs and SnRK2s) have also been implicated in ABA responses (Liu, 2012). The MKK9-MPK3/MPK6 cascade could promote ethylene-insensitive 3 (EIN3)-mediated transcription in ethylene signaling (Yoo et al., 2008). Furthermore, MAPK cascades were related to the regulation of auxin biosynthesis, transport, and signal transduction (Keithanne and Stephen, 2000; Smekalova et al., 2014). Notably, it has also been reported that the MAPK signaling pathway is involved in PCD (Li et al., 2007; Li and Franklin-Tong, 2011). In the present study, most DEGs were highly expressed in the M08 inbred line, particularly the ABA signal transduction-related elements, while ETH signaling genes were highly expressed in the M03 line (Figure 6B). Therefore, the MAPK signaling pathway may participate in adjusting pericarp thickness by regulating phytohormone-mediated PCD.

Pericarp thickness is an important factor affecting the filling rate. In sorghum, the differences in pericarp thickness between different varieties were related to the starch content in the mesocarp cells; thicker pericarp varieties contained more starch (Earp et al., 2004). Consistently, we found more starch in the pericarp cells of the M08 inbred line (thicker pericarp) than in those of the M03 line. The dynamic conversion of starch and sucrose in plant organs is necessary to maintain growth and development. Plant organ PCD is beneficial for nutrient reuse. The starch in pericarp cells is likely to be converted to sucrose because of pericarp cell PCD. During this process, ABA and GA play important roles in promoting starch degradation and sucrose remobilization (Moreno et al., 2011; Chen and Wang, 2012). Hexose sugars formed by the cleavage of sucrose via the action of invertases can be consumed during starch synthesis and by cell wall components (e.g., cellulose, hemicellulose, and lignin) (Koch, 2004). We speculate that during pericarp thinning, starch granules are degraded into sugars, such as sucrose, before entering the endosperm cells to resynthesize starch or are further converted into sugars required to synthesize the pericarp cell wall, causing the latter to thicken.

Terpenoids, also known as isoprenoids, have a basic skeleton composed of isoprene units. They are a class of natural hydrocarbon compounds widely occurring in the plant kingdom and have critical physiological functions. It is not primary metabolites that play an essential role in plant growth and development, but, rather, terpenoids as secondary metabolites (Quan, 2013), e.g., phytohormones GAs, BRs, and CKs, play a pivotal role (Tarkowska and Strnad, 2018). The chief among diterpenoid natural products is GA phytohormones (Yamaguchi, 2008). BRs are a group of plant signal molecules with a tetracyclic steroid skeleton derived from triterpenes related to plant growth regulation (Cano-Delgado et al., 2004). CKs are adenine derivatives that can be modified at the N6 position with either isoprenoid or aromatic side chains to yield isoprenoid CKs (ISCK) or aromatic CKs (ARCK), respectively (Tarkowska et al., 2014). Most of the DEGs were highly expressed in the M03 line. Similarly, most genes related to GA and BR signaling were also highly expressed in the M03 line. As previously described, all three hormone types induce PCD in plant cells. Therefore, we conjecture that the terpenoid skeleton may be involved in synthesizing phytohormones to regulate pericarp thickness and that GA and BR signaling seem to dominate in the M03 line.

Protein kinases are widespread in systems such as signaling transduction and cell cycle regulation (Satterlee and Sussman, 1998). Serine/threonine protein kinases are an important class of protein kinases whose function is to phosphorylate serine and threonine residues on proteins. “Glycine, serine, and threonine metabolism” provide substances for synthesizing serine/threonine protein kinases. Several serine/threonine protein kinases were found to be involved in plant hormone signal transduction (Marciniak et al., 2013). Studies suggest that heterodimerization between two serine/threonine protein receptors (BRI1 and BAK1) might be a key step in BR perception and signaling in plants (Li, 2003; Bojar et al., 2014). There are serine/threonine protein kinase elements in both ABA and ETH signal transduction pathways (Iten et al., 1999). For instance, ABI1 and SnRK2, two serine/threonine protein kinases, are part of the ABA core signaling pathway (Tajdel et al., 2016). CTR1, a serine/threonine kinase, is a negative regulator of the ETH response pathway (Kieber et al., 1993). These hormones, however, have been reported to activate PCD in plant cells. Notably, the autophagy-related gene *Atg1*, identified as serine/threonine protein kinase, appears to be essential in the early and late stages of autophagy (Kijanska and Peter, 2013). Most of the DEGs were highly expressed in the M03 line, suggesting that the synthesis of serine/threonine protein kinases may be more active in M03.

## Conclusion

**FIGURE 9:**
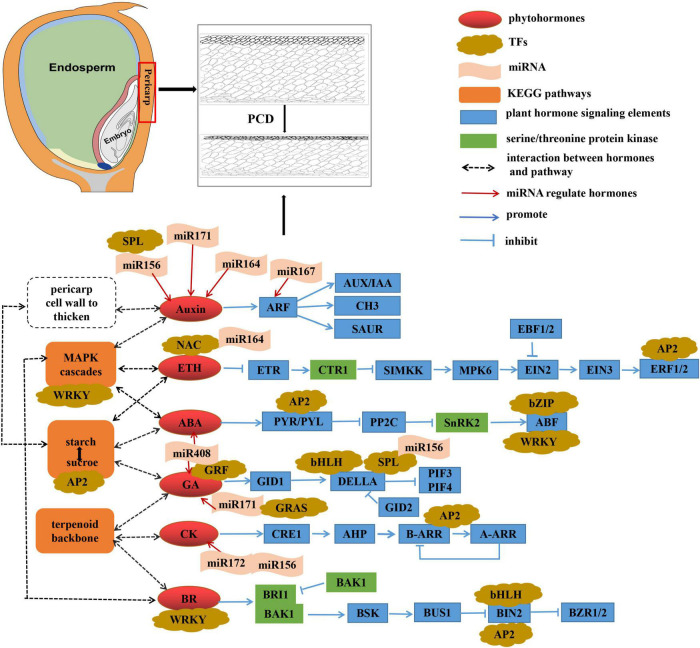
Hypothetical mechanisms regulating sweet corn pericarp thickness.

We identified five critical pathways related to pericarp thickness and specific miRNAs (such as zma-miR164, zma-miR167, zma-miR156, and zma-miR171) that may be involved in regulating pericarp thickness. We believe that plant hormone signal transduction plays a fundamental role in inducing changes in the pericarp thickness of sweet corn (Figure 9). The “MAPK signaling pathway” and “terpenoid backbone biosynthesis” may indirectly regulate changes in pericarp thickness in a way that participates in phytohormone synthesis. “Starch and sucrose metabolism” may obtain the carbohydrates required to synthesize cell walls during interconversion, and phytohormones can promote this process, thereby thickening the pericarp cell wall. Serine/threonine protein kinases play an important role in plant hormone signal transduction and can also induce autophagy. “Glycine, serine, and threonine metabolism” provides substances for synthesizing serine/threonine protein kinases. Furthermore, different sweet corn varieties have slightly distinct mechanisms for regulating pericarp thickness. AUX, GA, and BR signal transduction may indirectly mediate PCD to regulate pericarp thickness in the M03 line. In contrast, ABA, CK, and ETH signaling may be the key to regulating pericarp PCD in the M08 line. The results provide a reference for the genetic mechanism of pericarp thickness in sweet corn and reveal its nutritional value and flavor substance base.

## Data availability statement

The original contributions presented in this study are publicly available. This data can be found here: NCBI, PRJNA807465 and PRJNA807472.

## Author contributions

CX: conceptualization, methodology, investigation, visualization, and writing – original draft preparation. HP: software, investigation, and data curation. YZ and WR: investigation and data curation. ZM and YT: investigation. JH: supervision, funding acquisition, writing – original draft, and review and editing, and project administration. All authors have read and agreed to the published version of the manuscript.

## Footnotes

1
https://www.bioinformatics.babraham.ac.uk/projects/fastqc/

2
https://download.maizegdb.org/Zm-B73-REFERENCE-GRAMENE-4.0/Zm-B73-REFERENCE-GRAMENE-4.0.fa.gz

3
https://ccb.jhu.edu/software/hisat2/index.shtml

4
http://ccb.jhu.edu/software/stringtie/

5
http://bowtie-bio.sourceforge.net/bowtie2/index.shtml

6
https://www.arb-silva.de/

7
http://gtrnadb.ucsc.edu/

8
https://rfam.org/

9
http://www.girinst.org/Repbase_Update.html

10
http://bioconductor.org/packages/DEGseq/

11
http://targetfinder.org/

12
http://www.geneontology.org/

13
http://www.genome.jp/kegg/

## Abbreviations

ABA: abscisic acid
ARFs: auxin response factors
AUX: auxin
BR: brassinosteroid
CK: cytokinin
DAP: days after pollination
DEG: differentially expressed gene
DEMI: differentially expressed miRNA
KEGG: Kyoto Encyclopedia of Genes and Genomes
ETH: ethylene
GA: gibberellin
GO: Gene Ontology
MAPK: mitogen-activated protein kinase
PCD: programmed cell death
SEM: scanning electron microscopy.
